# Supplementary material for: Macronutrient Intake in Pregnancy and Child Cognitive and Behavioural Outcomes
Source: Children (Basel). 2021 May 20;8(5):425. doi: 10.3390/children8050425 (PMC8161020; doi:10.3390/children8050425)
Supplement: Supplementary file 1 [file children-08-00425-s001.zip › children-1201111-supplementary.pdf]

**Supplementary Material**

**Table S1.** Association of maternal dietary composition during pregnancy (not adjusted for energy intake) with child cognition outcomes up to age 4-years (*n*=58).

| Variables[1] | Beta-coefficient | 95% Confidence interval | P-value[2] | R-value |
|---|---|---|---|---|
| **Full scale IQ** | | | | |
| Energy | −1.12 | −9.45 to 7.21 | 0.79 | 0.15 |
| Protein (% E) | 6.85 | −16.49 to 30.20 | 0.56 | 0.15 |
| Total fat (% E) | 5.34 | −13.82 to 24.49 | 0.58 | 0.15 |
| PUFA (% E) | 0.32 | −7.73 to 8.37 | 0.94 | 0.15 |
| CHO (% E) | −12.65 | −35.10 to 9.80 | 0.26 | 0.17 |
| P:C ratio | −0.83 | −6.75 to 5.10 | 0.78 | 0.15 |
| Protein (g) | −0.23 | −8.20 to 7.74 | 0.95 | 0.15 |
| PUFA (g) | −0.41 | −6.49 to 5.66 | 0.89 | 0.15 |
| Total sugars (g) | −0.27 | −7.53 to 6.99 | 0.94 | 0.15 |
| Starch (g) | −4.69 | −12.42 to 3.04 | 0.23 | 0.17 |
| **Verbal IQ** | | | | |
| Energy | 1.28 | −9.72 to 12.29 | 0.82 | 0.04 |
| Protein (% E) | 0.49 | −30.44 to 31.42 | 0.98 | 0.04 |
| Total fat (% E) | 11.21 | −13.88 to 36.30 | 0.38 | 0.06 |
| PUFA (% E) | −0.40 | −10.91 to 10.11 | 0.94 | 0.04 |
| CHO (% E) | −13.10 | −42.79 to 16.59 | 0.38 | 0.06 |
| P:C ratio | −2.87 | −10.66 to 4.92 | 0.46 | 0.05 |
| Protein (g) | 1.23 | −9.29 to 11.75 | 0.82 | 0.04 |
| PUFA (g) | 0.45 | −7.53 to 8.42 | 0.91 | 0.04 |
| Total sugars (g) | 1.32 | −8.27 to 10.91 | 0.78 | 0.04 |
| Starch (g) | −2.50 | −12.81 to 7.81 | 0.63 | 0.05 |
| **Performance IQ** | | | | |
| Energy | −4.52 | −13.93 to 4.88 | 0.34 | 0.20 |
| Protein (% E) | 14.96 | −11.36 to 41.28 | 0.26 | 0.20 |
| Total fat (% E) | 2.26 | −19.60 to 24.10 | 0.84 | 0.18 |
| PUFA (% E) | −1.82 | −10.94 to 7.31 | 0.69 | 0.18 |
| CHO (% E) | −14.59 | −40.13 to 10.96 | 0.26 | 0.20 |
| P:C ratio | 1.21 | −5.52 to 7.95 | 0.72 | 0.18 |
| Protein (g) | −2.40 | −11.43 to 6.64 | 0.60 | 0.19 |
| PUFA (g) | −3.44 | −10.29 to 3.41 | 0.32 | 0.20 |
| Total sugars (g) | −2.98 | −11.18 to 5.22 | 0.47 | 0.19 |
| **Starch (g)** | **−8.06** | **−16.70 to 0.57** | **0.07** | **0.23** |
| **Processing speed composite** | | | | |
| Energy | −0.01 | −9.06 to 9.03 | 0.99 | 0.18 |
| Protein (% E) | 6.37 | −16.25 to 28.99 | 0.57 | 0.19 |

| | | | | |
|---|---|---|---|---|
| Total fat (% E) | 1.03 | −17.69 to 19.74 | 0.91 | 0.18 |
| PUFA (% E) | −1.01 | −8.66 to 6.64 | 0.79 | 0.18 |
| CHO (% E) | −8.33 | −30.26 to 13.61 | 0.45 | 0.19 |
| P:C ratio | 3.60 | −2.01 to 9.21 | 0.20 | 0.21 |
| PUFA (g) | −0.67 | −6.89 to 5.54 | 0.82 | 0.18 |
| Protein (g) | 0.82 | −7.40 to 9.05 | 0.84 | 0.18 |
| Total sugars (g) | 0.50 | −6.89 to 7.89 | 0.89 | 0.18 |
| Starch (g) | −3.78 | −12.99 to 5.43 | 0.41 | 0.19 |

**Table S1.** *(Continued)*

| **Variable**[1] | **Beta-coefficient** | **95% Confidence interval** | **P-value**[2] | **R- value** |
|---|---|---|---|---|
| **General language composite** | | | | |
| Energy | 2.36 | −10.55 to 15.26 | 0.72 | 0.06 |
| Protein (%E) | 8.40 | −27.84 to 44.63 | 0.64 | 0.06 |
| Total fat (% E) | 14.82 | −14.57 to 44.22 | 0.32 | 0.07 |
| PUFA (% E) | 2.78 | −9.54 to 15.10 | 0.65 | 0.06 |
| CHO (% E) | −19.55 | −54.25 to 15.15 | 0.26 | 0.08 |
| P:C ratio | −0.69 | −9.88 to 8.49 | 0.88 | 0.06 |
| Protein (g) | 3.13 | −9.19 to 15.45 | 0.61 | 0.06 |
| PUFA (g) | 2.84 | −6.49 to 12.17 | 0.55 | 0.06 |
| Total sugars (g) | 2.44 | −8.80 to 13.68 | 0.67 | 0.06 |
| Starch (g) | −3.62 | −15.71 to 8.47 | 0.55 | 0.06 |

CHO, carbohydrates, P:C, protein to carbohydrate, PUFA, polyunsaturated fatty acids, % E, percentage of energy. Analysis models were adjusted for maternal age, education, pre-pregnancy BMI and birthweight. [1] The natural logarithm transformation of the nutrient variable was used for the linear regression models to meet normality assumptions. [2] P-values were derived by linear regression models.

**Table S2.** Association of maternal dietary composition during pregnancy (not adjusted for energy intake) with child behaviour outcomes up to age 4-years (*n*=51)

| **Variables[1]** | **Beta-coefficient** | **95% Confidence interval** | **P-value[2]** | **R-value** |
|---|---|---|---|---|
| **Total problems score** | | | | |
| Energy | 13.94 | −11.82 to 39.70 | 0.28 | 0.22 |
| Protein (% E) | 0.71 | −72.44 to 73.87 | 0.98 | 0.20 |
| PUFA (% E) | −13.21 | −40.05 to 13.64 | 0.33 | 0.21 |
| Total fat (% E) | 3.36 | −61.98 to 68.70 | 0.92 | 0.20 |
| CHO (% E) | 14.06 | −62.11 to 90.23 | 0.71 | 0.21 |
| P:C ratio | 7.46 | −11.49 to 26.42 | 0.43 | 0.21 |
| Protein (g) | 12.65 | −11.84 to 37.14 | 0.30 | 0.21 |
| PUFA (g) | 0.94 | −18.28 to 20.15 | 0.92 | 0.20 |
| Total sugars (g) | 0.35 | −19.27 to 53.27 | 0.35 | 0.21 |
| Starch (g) | 9.62 | −15.72 to 34.97 | 0.45 | 0.21 |
| **Internalising broad band score** | | | | |
| Energy | 3.12 | −22.54 to 28.78 | 0.81 | 0.27 |
| Protein (% E) | −6.20 | −78.23 to 65.84 | 0.86 | 0.27 |
| Total fat (% E) | 14.03 | −50.20 to 78.27 | 0.66 | 0.27 |
| PUFA (% E) | −13.55 | −39.96 to 12.87 | 0.31 | 0.28 |
| CHO (% E) | 1.69 | −73.42 to 76.82 | 0.96 | 0.27 |
| P:C ratio | 9.23 | −9.37 to 27.83 | 0.32 | 0.28 |
| Protein (g) | 2.10 | −22.27 to 26.48 | 0.86 | 0.27 |
| PUFA (g) | −5.11 | −23.98 to 13.76 | 0.59 | 0.27 |
| Total sugars (g) | 5.52 | −17.28 to 28.33 | 0.63 | 0.27 |
| Starch (g) | 1.58 | −23.52 to 26.69 | 0.90 | 0.27 |

| **Externalising broad band score** | | | | |
|---|---|---|---|---|
| Energy | 20.03 | −5.35 to 45.42 | 0.12 | 0.19 |
| Protein (% E) | 1.28 | −71.75 to 74.31 | 0.97 | 0.15 |
| Total fat (% E) | 14.04 | −51.07 to 79.15 | 0.67 | 0.15 |
| PUFA (% E) | −12.04 | −38.88 to 14.80 | 0.37 | 0.16 |
| CHO (% E) | −2.44 | −79.96 to 75.07 | 0.95 | 0.10 |
| P:C ratio | 5.83 | −13.14 to 24.80 | 0.54 | 0.15 |
| Protein (g) | 18.21 | −5.95 to 42.36 | 0.14 | 0.19 |
| PUFA (g) | 4.83 | −14.30 to 23.96 | 0.61 | 0.15 |
| Total sugars (g) | 21.41 | −0.93 to 43.75 | 0.06 | 0.21 |
| Starch (g) | 13.75 | −11.39 to 38.89 | 0.28 | 0.17 |

CHO, carbohydrates, P:C, protein to carbohydrate, PUFA, polyunsaturated fatty acids, % E, percentage of energy. Analysis models were adjusted for maternal age, education, pre-pregnancy BMI, birthweight and breastfeeding duration (weeks). [1] The natural logarithm transformation of the nutrient variable was used for the linear regression models to meet normality assumptions. [2] P-values were derived by linear regression models.

**Table S3.** Association of maternal dietary composition during pregnancy with child cognition outcomes up to age 4-years (*n*=58) without adjustment for covariates

| Variables[1] | Beta-coefficient | 95% Confidence interval | P-value[2] | R-value |
|---|---|---|---|---|
| **Full scale IQ** | | | | |
| Energy | −3.49 | −13.63 to 6.65 | 0.49 | 0.01 |
| Protein (% E) | 6.23 | −17.52 to 29.98 | 0.60 | 0.00 |
| Total fat (% E) | −0.01 | −21.96 to 21.92 | 1.00 | 0.00 |
| PUFA (% E) | 0.32 | −8.08 to 8.72 | 0.94 | 0.00 |
| CHO (% E) | −5.10 | −29.78 to 19.57 | 0.68 | 0.00 |
| P:C ratio | −1.99 | −8.16 to 4.18 | 0.52 | 0.01 |
| Protein (g) | −2.06 | −11.56 to 7.45 | 0.67 | 0.00 |
| PUFA (g) | −1.49 | −8.63 to 5.65 | 0.68 | 0.00 |
| Total sugars (g) | −0.48 | −8.44 to 7.49 | 0.91 | 0.00 |
| Starch (g) | −6.47 | −15.61 to 2.67 | 0.16 | 0.03 |
| **Verbal IQ** | | | | |
| Energy | 1.81 | −10.73 to 14.35 | 0.77 | 0.00 |
| Protein (% E) | 1.84 | −9.90 to 13.57 | 0.76 | 0.00 |
| Total fat (% E) | 10.20 | −16.32 to 36.72 | 0.77 | 0.01 |
| PUFA (% E) | 1.11 | −9.17 to 11.40 | 0.83 | 0.00 |
| CHO (% E) | −11.00 | −41.04 to 19.02 | 0.47 | 0.00 |
| P:C ratio | −2.73 | −10.34 to 4.88 | 0.48 | 0.01 |
| Protein (g) | 1.84 | −9.90 to 13.57 | 0.76 | 0.00 |
| PUFA (g) | 1.71 | −7.08 to 10.50 | 0.70 | 0.00 |
| Total sugars (g) | 2.05 | −7.77 to 11.87 | 0.68 | 0.00 |
| Starch (g) | −2.98 | −14.43 to 8.47 | 0.60 | 0.00 |
| **Performance IQ** | | | | |
| Energy | −7.22 | −18.82 to 4.39 | 0.22 | 0.03 |
| Protein (% E) | 9.12 | −18.27 to 36.51 | 0.51 | 0.01 |
| Total fat (% E) | −1.14 | −26.43 to 24.15 | 0.93 | 0.00 |
| PUFA (% E) | −2.86 | −12.50 to 6.77 | 0.55 | 0.01 |
| CHO (% E) | −6.82 | −35.28 to 21.64 | 0.63 | 0.00 |
| P:C ratio | −1.16 | −8.31 to 5.98 | 0.75 | 0.00 |
| Protein (g) | −4.85 | −15.80 to 6.07 | 0.38 | 0.01 |
| PUFA (g) | −5.62 | −13.73 to 2.48 | 0.17 | 0.03 |
| Total sugars (g) | −3.55 | −12.71 to 5.60 | 0.44 | 0.01 |
| Starch (g) | −9.06 | −19.49 to 1.38 | 0.09 | 0.05 |
| **Processing speed composite** | | | | |
| Energy | −0.17 | −11.30 to 10.95 | 0.98 | 0.00 |
| Protein (% E) | 6.26 | −17.74 to 30.26 | 0.60 | 0.00 |
| Total fat (% E) | −3.63 | −25.45 to 18.19 | 0.74 | 0.00 |
| PUFA (% E) | −0.62 | −7.95 to 6.71 | 0.87 | 0.00 |

| | | | | |
|---|---|---|---|---|
| CHO (% E) | −2.96 | −27.33 to 21.40 | 0.81 | 0.00 |
| P:C ratio | 1.47 | −4.57 to 7.50 | 0.63 | 0.00 |
| PUFA (g) | −0.62 | −7.95 to 6.71 | 0.87 | 0.00 |
| Protein (g) | 0.87 | −8.77 to 10.51 | 0.86 | 0.00 |
| Total sugars (g) | 0.91 | −7.05 to 8.86 | 0.82 | 0.00 |
| Starch (g) | 5.66 | −14.88 to 7.83 | 0.54 | 0.01 |
| **Table S3.** *(Continued)* | | | | |
| **Variable[1]** | **Beta-coefficient** | **95% Confidence interval** | **P-value[2]** | **R- value** |
| **General language composite** | | | | |
| **Energy** | 0.78 | −13.78 to 15.34 | 0.92 | 0.00 |
| **Protein (%E)** | 10.48 | −23.49 to 44.47 | 0.54 | 0.00 |
| **Total fat (% E)** | 8.45 | −22.41 to 39.30 | 0.59 | 0.00 |
| **PUFA (% E)** | 4.19 | −7.70 to 16.08 | 0.48 | 0.00 |
| **CHO (% E)** | −12.59 | −47.45 to 22.26 | 0.47 | 0.01 |
| **P:C ratio** | −1.29 | −10.15 to 7.58 | 0.78 | 0.00 |
| **Protein (g)** | 2.36 | −11.25 to 15.97 | 0.73 | 0.00 |
| **PUFA (g)** | 3.45 | −6.73 to 13.62 | 0.50 | 0.00 |
| **Total sugars (g)** | 2.98 | −8.41 to 14.37 | 0.60 | 0.00 |
| **Starch (g)** | −6.72 | −19.92 to 6.48 | 0.31 | 0.00 |

CHO, carbohydrates, P:C, protein to carbohydrate, PUFA, polyunsaturated fatty acids, % E, percentage of energy. Analysis models were adjusted for maternal age, education, pre-pregnancy BMI and birthweight. [1] The natural logarithm transformation of the nutrient variable was used for the linear regression models to meet normality assumptions. [2] P-values were derived by linear regression models.

**Table S4.** Association of maternal dietary composition during pregnancy with child behaviour outcomes up to age 4-years (*n*=51) without adjustment for covariates

| Variables[1] | Beta-coefficient | 95% Confidence interval | P-value[2] | R-value |
|---|---|---|---|---|
| **Total problems score** | | | | |
| Energy | 20.86 | −10.58 to 52.31 | 0.19 | 0.03 |
| Protein (% E) | −23.42 | −95.72 to 48.87 | 0.52 | 0.01 |
| PUFA (% E) | −15.24 | −42.48 to 12.01 | 0.27 | 0.02 |
| Total fat (% E) | 31.91 | −41.01 to 104.83 | 0.38 | 0.02 |
| CHO (% E) | −2.01 | −86.73 to 82.71 | 0.96 | 0.00 |
| P:C ratio | 2.91 | −17.15 to 22.97 | 0.77 | 0.00 |
| Protein (g) | 13.95 | −15.37 to 43.27 | 0.34 | 0.02 |
| PUFA (g) | 0.18 | −22.23 to 22.58 | 0.99 | 0.00 |
| Total sugars (g) | 16.41 | −8.26 to 41.09 | 0.19 | 0.03 |
| Starch (g) | 16.87 | −14.33 to 48.07 | 0.28 | 0.02 |
| **Internalising broad band score** | | | | |
| Energy | 5.50 | −26.67 to 37.68 | 0.73 | 0.00 |
| Protein (% E) | −37.40 | −109.71 to 34.91 | 0.30 | 0.02 |
| Total fat (% E) | −12.17 | −39.72 to 15.38 | 0.40 | 0.02 |
| PUFA (% E) | 43.58 | −29.35 to 116.51 | 0.24 | 0.03 |
| CHO (% E) | −12.86 | −98.07 to 72.35 | 0.76 | 0.00 |
| P:C ratio | 2.31 | −17.88 to 22.51 | 0.82 | 0.00 |
| Protein (g) | 10.32 | −31.29 to 28.28 | 0.92 | 0.00 |
| PUFA (g) | 11.20 | −27.83 to 17.17 | 0.64 | 0.00 |
| Total sugars (g) | 3.56 | −21.70 to 28.82 | 0.78 | 0.00 |
| Starch (g) | 4.91 | −26.83 to 36.66 | 0.76 | 0.00 |

| | | | | |
|---|---|---|---|---|
| **Externalising broad band score** | | | | |
| Energy | 28.23 | −2.30 to 58.76 | 0.07 | 0.06 |
| Protein (% E) | −15.05 | −86.55 to 56.45 | 0.67 | 0.00 |
| Total fat (% E) | 32.06 | −39.86 to 103.99 | 0.38 | 0.02 |
| PUFA (% E) | −17.72 | −44.46 to 9.03 | 0.19 | 0.19 |
| CHO (% E) | −7.26 | −90.83 to 76.30 | 0.87 | 0.00 |
| P:C ratio | 2.17 | −17.62 to 21.97 | 0.83 | 0.00 |
| Protein (g) | 21.64 | −6.90 to 50.18 | 0.13 | 0.04 |
| PUFA (g) | −17.72 | −44.46 to 9.03 | 0.19 | 0.03 |
| Total sugars (g) | 20.77 | −3.29 to 44.84 | 0.09 | 0.06 |
| Starch (g) | 21.95 | −8.56 to 52.46 | 0.16 | 0.04 |

CHO, carbohydrates, P:C, protein to carbohydrate, PUFA, polyunsaturated fatty acids, % E, percentage of energy. Analysis models were adjusted for maternal age, education, pre-pregnancy BMI, birthweight and breastfeeding duration (weeks). [1] The natural logarithm transformation of the nutrient variable was used for the linear regression models to meet normality assumptions. [2] P-values were derived by linear regression models.
